# Flavor Compounds in Pixian Broad-Bean Paste: Non-Volatile Organic Acids and Amino Acids

**DOI:** 10.3390/molecules23061299

**Published:** 2018-05-29

**Authors:** Hongbin Lin, Xiaoyu Yu, Jiaxing Fang, Yunhao Lu, Ping Liu, Yage Xing, Qin Wang, Zhenming Che, Qiang He

**Affiliations:** 1 College of Light Industry and Food Engineering, Sichuan University, Chengdu 610065, China; hongbin-ok@163.com (H.L.); luyunhao@foxmail.com (Y.L.); 2 College of Food and Bio-engineering, Xihua University, Chengdu 610039, China; yxy93115@126.com (X.Y.); fangjx1994@126.com (J.F.); dewflowerlp@163.com (P.L.); xingyage1@163.com (Y.X.); celery11@hotmail.com (Q.W.); chezhenming@163.com (Z.C.); 3 Department of Nutrition and Food Science, Maryland University, College Park, MD 20742, USA

**Keywords:** non-volatile organic acid, amino acid, HPLC, amino acid automatic analyzer, Pixian broad-bean paste

## Abstract

Non-volatile organic acids and amino acids are important flavor compounds in Pixian broad-bean paste, which is a traditional Chinese seasoning product. In this study, non-volatile organic acids, formed in the broad-bean paste due to the metabolism of large molecular compounds, are qualitatively and quantitatively determined by high-performance liquid chromatography (HPLC). Amino acids, mainly produced by hydrolysis of soybean proteins, were determined by the amino acid automatic analyzer. Results indicated that seven common organic acids and eighteen common amino acids were found in six Pixian broad-bean paste samples. The content of citric acid was found to be the highest in each sample, between 4.1 mg/g to 6.3 mg/g, and malic acid were between 2.1 mg/g to 3.6 mg/g ranked as the second. Moreover, fumaric acid was first detected in fermented bean pastes albeit with a low content. For amino acids, savory with lower sour taste including glutamine (Gln), glutamic acid (Glu), aspartic acid (Asp) and asparagines (Asn) were the most abundant, noted to be 6.5 mg/g, 4.0 mg/g, 6.4 mg/g, 4.9 mg/g, 6.2 mg/g and 10.2 mg/g, and bitter taste amino acids followed. More importantly, as important flavor materials in Pixian broad-bean paste, these two groups of substances are expected to be used to evaluate and represent the flavor quality of Pixian broad-bean paste. Moreover, the results revealed that citric acid, glutamic acid, methionine and proline were the most important flavor compounds. These findings are agreat contribution for evaluating the quality and further assessment of Pixian broad-bean paste.

## 1. Introduction

Soybean pastes as traditional fermented products originated from East Asia with different names (because they are from different countries: China, Korea, Japan). Due to the unique taste and various health benefits of soybean paste, such as anticancer, antihypertension, immunity, and antioxidative activity [1,2], consumption of the fermented soybean products is gradually expanding to the whole world [3]. As one kind of soybean paste and a traditional Chinese fermented product, Pixian broad-bean pastes produced from chili (Two Vitex), steamed broad-bean, salt, and wheat flour and usually ripened for one year to three years. The quality and taste of commercial soybean paste is mainly dependent on the initial ingredients, the process of fermentation and microorganisms [4]. Flavor is one of key factors to evaluate a seasoning. The volatile compounds in Pixian broad-bean paste have been extensively studied [5,6,7]. However, the taste compounds have not been systematically researched. The reason seems to be obvious that the flavor of soybean paste is contributed by various compounds and they are difficult to be separated and analyzed. The water-soluble fraction of traditional Doenjang (a soybean paste from Korea) was found to contain a group of taste compounds, such as salts, amino acids, organic acids, and peptides, produced during proteolysis [8]. In addition, several studies showed that the characteristic flavors were derived from hydrolysis of proteins by microorganisms such as molds, yeasts, lactic acid bacteria [4]. Therefore, it is well-known that organic acids and amino acids are important flavor contents in the fermented products [9,10]. However, to the best of our knowledge, there is no report on the non-volatile organic acids, and amino acids composition of Pixian broad-bean paste. Therefore, the objective of this study is to systematically investigate non-volatile organic acids, and amino acids in six Pixian broad-bean paste samples, as the important flavor materials.

Organic acid compounds in various fermented soybean products have been studied extensively, such as Japanese miso and natto, Chinese sufu and Thai thuanao [11,12]. Organic acids occur in fermented products as a result of fermentation, hydrolysis, and microbial activity [13]. Because the organic acids contribute to the nutrition and unique taste of fermented foods, the qualitative and quantitative determination of organic acids is important for fermented foods [14]. The commonly used methods for the determination of acid content in food are titrimetric methods, gas chromatography, colorimetric analysis and high-performance liquid chromatography. However, the titration method and colorimetric analysis are not preferred because it is hard to accurately determine the type and content of the acids [15]. To avoid these problems, gas chromatography (GC) and high-performance liquid chromatography (HPLC) methods have been used in the organic acid analysis [14,16]. HPLC is generally preferred because of its speed, selectivity, sensitivity, reliability and simple sample preparation.

The unique flavor and taste of Doenjang were found to come from the decomposing product of soybean proteins, which was responded by the action of microorganisms [17]. The fermentation process affected the capacity of nutritional and functional properties compared to original products [18]. Amino acids were mainly produced by hydrolysis of soybean proteins, which contribute to the flavor characteristics of soybean products and also have an important effect on the consumer acceptance [19]. More specifically, the soybean protein was hydrolyzed to smaller nitrogen compounds such as peptides, amino acids, amines and ammonia [20], resulting in flavor enhancement. Therefore, it is important to analyze amino acids in fermented foods qualitatively and quantitatively. Currently, widely used methods for the determination of amino acid contents are high-performance liquid chromatography (HPLC) and amino acid automatic analyzer. Because of its preferred speed, selectivity and reliability, amino acid automatic analyzer is the most commonly used [19].

Non-volatile organic acids, and amino acids, as the important flavor materials in Pixian broad-bean paste, played an important role on the compliance of the product quality standards. Therefore, the objective of the present study is to analyze the non-volatile organic acids, and amino acids, by using HPLC and amino acid automatic analyzer, respectively, qualitatively and quantitatively. That is, individual non-volatile organic acids, and amino acids were qualitatively determined by being respectively compared with standard chromatograms and quantitatively determined by calculation with a standard curve. Finally, partial least squares regression (PLSR) was applied to analyze sensory evaluation in order to find the key flavor compounds in the Pixian broad-bean paste.

## 2. Results and Discussion

### 2.1. Determination of Non-Volatile Organic Acids

**Figure 1:**
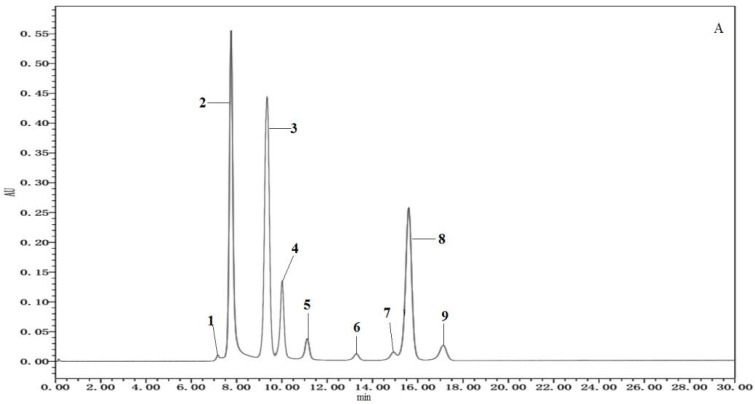

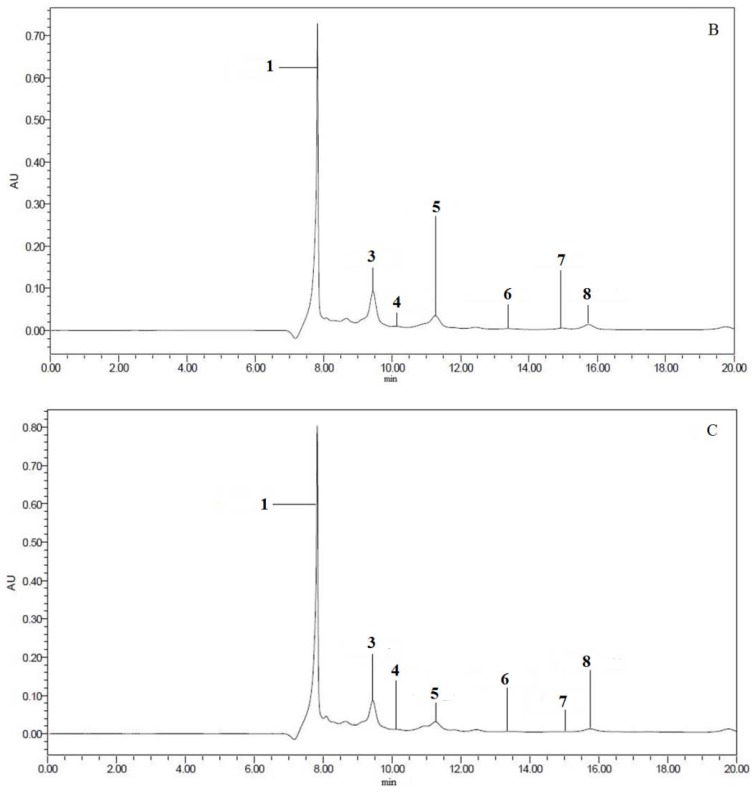
Results for organic acid analysis: (**A**) Standard solution of seven organic acids (1: Oxalic acid; 2: Ketoglutaric acid; 3: Citric acid; 4: Tartaric acid; 5: Malic acid; 6: Succinic acid; 7: Lactic acid; 8: Fumaric acid; 9: Adipic acid); (**B**) Sample 1; (**C**) Sample 2.

The organic acids have a substantial effect on the balance of the flavor, but also influence the chemical stability, pH, and the sample quality [21]. Individual organic acid standard and mixed standard were chromatographed separately, and the retention time and response of each organic acid were determined (Table 1). The individual organic acid standard was used to locate the retention time. The standard curve for quantitative testing was formed by chromatographing different concentrations of mixed organic acids. The compositions of organic acids of six samples were qualitatively determined by comparing with retention time of standards (Figure 1) and quantitatively determined by calculation with a standard curve (Table 1). The retention time of oxalic acid, ketoglutaric acid, citric acid, tartaric acid, malic acid, succinic acid, lactic acid, fumaric acid and adipic acid was 7.7 min, 8.2 min, 9.3 min, 10 min, 11.1 min, 13.2 min, 14.9 min, 15.6 min, 17.1 min, respectively.

The composition of organic acids in each sample is shown in Table 2. A total of nine organic acids, including oxalic acid, succinic acid, fumaric acid, lactic acid, ketoglutaric acid, tartaric acid, malic acid, adipic acid and citric acid, were detected. However, result indicated that adipic acid was not detected in any samples, and ketoglutaric acid was found in sample 6 only. Simultaneously, fumaric acid was first detected in fermented bean pastes although the content was much lower than the other organic acids. The other six acids, as the main organic acids were identified in Pixian broad-bean paste samples based on the analysis. The content of total organic acids in six samples was ranged from 9.2781 mg/g to 12.3643 mg/g Pixian broad-bean paste on wet base. Choi and Kim compared organic acid contents of three Korean soybean sauce produced from different raw materials (non-germinated meju (MNG), germinated soybeans under light (MGL) and dark (MGD)). They found that total amount of six organic acids (tartaric acid, malic acid, lactic acid, acetic acid, citric acid and succinic acid), was (9.422 ± 0.7 mg/g, MNG), (10.756 ± 0.8 mg/g, MGL), (10.197 ± 0.8 mg/g, MGD) [22]. Another study found that the fermentation environment, the microbial population and the origin of raw soybeans had important effects on the organic acid content in the soybean paste [23]. 

As can be seen in Table 2, results revealed that citric acid content was highest in all six Pixian broad-bean samples while comparing to other organic acids, which is consistent with what Park et al. has reported. The citric acid in different samples were found to be 4.8283 mg/g, 6.3000 mg/g, 5.1342 mg/g, 5.2523 mg/g, 4.1978 mg/g and 4.3645 mg/g for samples 1–6, respectively. Citric acid, as one of the flavor content to be added to adjust the acidity, has the advantage of not forming insoluble precipitates with calcium and potassium [24]. Organic acids in the soybean paste, including citric acid, came from chili or bacteria metabolism in the late fermentation [25]. Citric acid is produced commercially by submerged fermentation using Aspergillus niger, and its applications include acidulation and flavor enhancement [26,27]. Moreover, Sunitha used response surface method (RSM) to optimize the correlative microbial systems [28]. While the malic acid contents were between 2.1 to 3.6 mg/g ranked as the second, and the oxalic acid contents were between 1.7 to 2.4 mg/g. Ketoglutaric acid was detected at 0.33 mg/g only in sample 6. Similar to the findings in a commercial soybean paste from Park, most of common non-volatile organic acids were detected in all six Pixian broad-bean paste samples. However, in the study, no malonic acid and glutaric acid were detected, because sample type is different from that used.

### 2.2. Determination of Amino Acids

**Figure 2:**
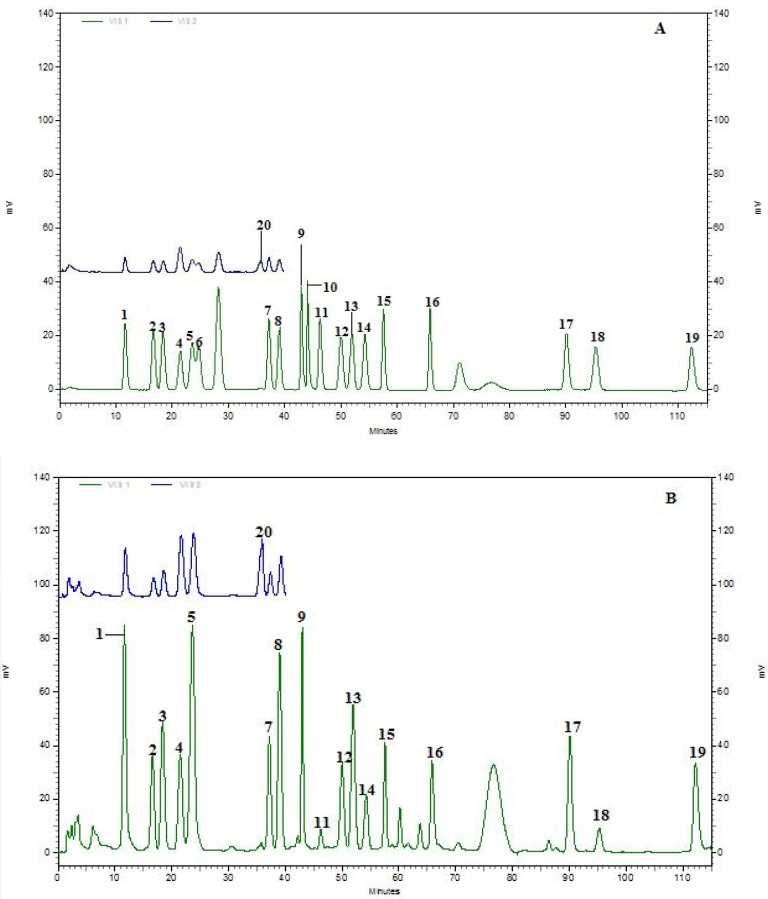

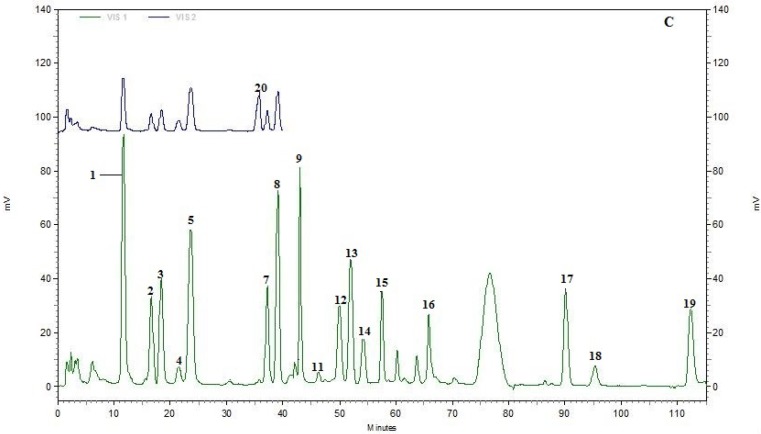
Results for amino acid analysis: (**A**) Standard solution of twenty amino acid (1: Asp; 2: Thr; 3: Ser; 4: Asn; 5: Glu; 6: Gln; 7: Gly; 8: Ala; 9: Val; 10: Cys; 11: Met; 12: Ile; 13: Leu; 14: Tyr; 15: Phe; 16: g-ABA; 17: Lys; 18: His; 19: Arg; 20: Pro); (**B**) Sample 1; (**C**) Sample 2.

Amino acid contents in fermented samples of Pixian broad-bean paste were determined by the amino acid automatic analyzer. Different concentrations of the amino acid standard were chromatographed, which were used to form a standard curve for quantitative testing. The individual amino acids of six samples were qualitatively and quantitatively determined by comparison with standard chromatograms (Figure 2). Meanwhile, referring to Park et al., through the analysis of different taste of amino acids, amino acids were divided into five categories [13], namely showing sweet with savory taste, savory with sour taste, bitter with sweet taste, bitter taste and salty taste (Table 3). Different amino acids contributed to the different category taste. The first class was tasted sweet with lower savory (threonine (Thr), serine (Ser), glycine (Gly), alanine (Ala) and proline (Pro)), the second class was tasted savory with sour (glutamine (Gln), glutamic acid (Glu), aspartic acid (Asp) and asparagine (Asn), the third was tasted bitter with lower savory (lysine (Lys), histidine (His) and arginine (Arg), the forth class was only tasted bitter (methionine (Met), isoleucine (Ile), leucine (Leu), valine (Val), tyrosine (Tyr), phenylalanine (Phe)), and the last class was cysteine (Cys), which tasted salty.

Results indicated that a total of twenty amino acids, including eighteen amino acids and two amides in each sample, were analyzed from Table 4. However, the compounds ofglutamine (Gln) and cysteine (Cys) were not detected in any samples. Meanwhile, the rest of the eighteen amino acids which were compared and analyzed were in the samples. Most of amino acids occur in Pixian broad-bean paste as a result of microbial activity and proteolysis. Moreover, some changes in amino acid content can be detected during the fermentation process. Studies have shown that most of the free amino acid contents during the fermentation process increased about 60 times [18]. On the other hand, some of amino acids were decreased, because the degree of soy proteins metabolized was greater than proteolysis [29].

In this work, the most abundant of amino acids with savory with the sour taste was detected in these six samples. According to the result in Table 4, the total amount of the four amino acids was noted to be 6.5 mg/g, 4.0 mg/g, 6.4 mg/g, 4.9 mg/g, 6.2 mg/g and 10.2 mg/g Pixian broad-bean paste on wet base. Kim and Rhee found that glutamic acid, aspartic acid in Doenjang samples had savory taste, which results from the hydrolysis of soybean proteins [30]. Han and Frans found that glutamic acid was the most abundant amino acid, followed by aspartic acid, representing together around 30% in sufu [18]. The results were consistent with earlier reports, among those six samples, half of them had the highest content of glutamic acid, and the content of the aspartic acid in the other three were the highest. More interestingly, both of the two amino acids had savory with lower sour flavor. The glutamic acid in combination with salt (NaCl) could provide the flavor appealing to the consumers [31]. Bitter taste and sweet with savory taste amino acids, ranked as the second and the third, were noted to be 4.4 mg/g, 3.9 mg/g, 6.0 mg/g, 4.1 mg/g, 4.5 mg/g, 11.1 mg/g and 5.1 mg/g, 3.9 mg/g, 5.5 mg/g, 4.0 mg/g, 4.9 mg/g, 9.9 mg/g for samples 1-6, respectively. Similar findings were observed that threonine (Thr), serine (Ser), glycine (Gly), alanine (Ala) amino acids contributed to the sweet taste of Doenjang samples [32]. In addition, there was a small amount of bitter with lower sweet amino acid ingredients in the six samples, and types of amino acids may vary depending on the sample type. 

### 2.3. Relationship between Pixian Broad-Bean Paste Samples, Organic Acids, Amino Acids and Sensory Attributes

**Figure 3:**
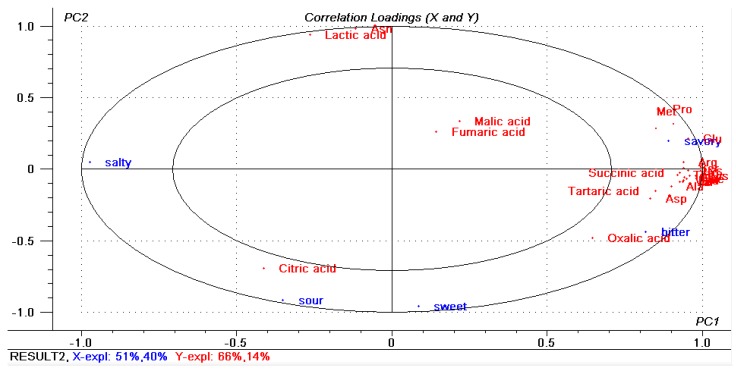
The overview of the variation found in the mean data from the partial least squares regression (PLSR) correlation loadings plot for Pixian broad-bean paste samples. The model was derived from sensory attributes as the X-matrix and taste-active compounds as Y-matrix. Circles represent *r*^2^ = 0.5 and 1.0, respectively.

**Figure 4:**
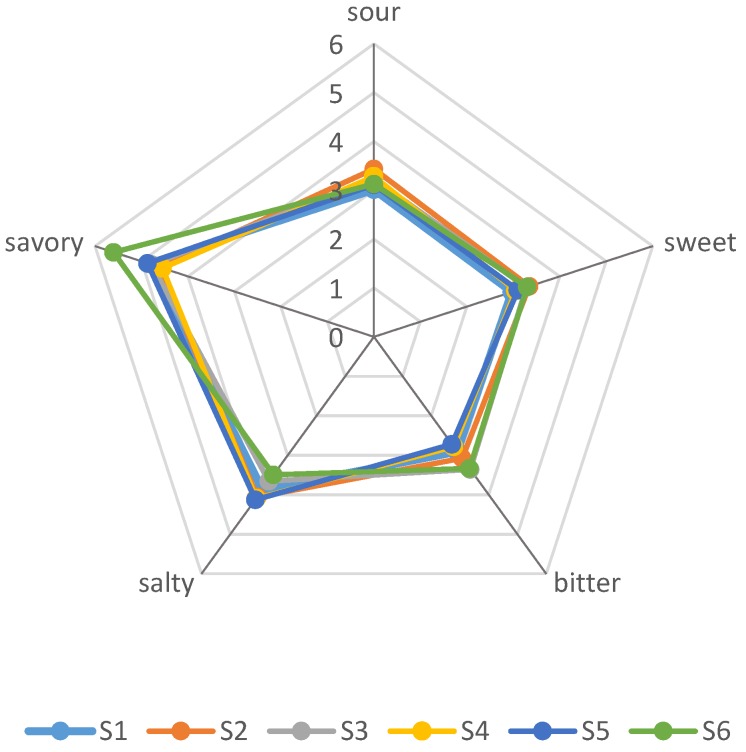
Graph of the sensory analysis of the Pixian broad-bean paste samples.

ANOVA-PLSR was used to process the mean data accumulated from organic acids, amino acids and sensory evaluation by the panelists [33]. 24 taste-active compounds were used as variables in the subsequent PLSR analysis. As shown in Figure 3, the X-matrix was designed as sensory variables, and the Y-matrix was designed as samples taste-active compounds. The calibrated explained variance for this model was PC1 = 51% and PC2 = 40%. It was presented the correlation loadings plot that the big and small circles indicated 50% and 100% explained variances, respectively [33]. Almost all the compounds except malic acid and Fumaric acid were placed between the inner and outer ellipses, indicating they were well explained by the PLSR model. Results indicated that sour taste was associated with citric acid and bitter taste was correlative with oxalic acid. Compared with other samples, as revealed in Figure 4, sample 2 had the most citric acid and it had the highest score in sour taste, which can confirm the above point of view. Meanwhile, savory taste was in the right upper hand quadrant and correlated with much of amino acids especially glutamic acid, methionine and proline. This was in accordance with the sensory evaluation results (Figure 4). While sample6 contained the most glutamic acid, methionine and proline comparing with other samples, and showed the highest score in savory taste. For instance, citric acid, glutamic acid, methionine and proline can be regarded as the most important flavor compounds in the Pixian broad-bean paste.

## 3. Materials and Methods

### 3.1. Materials

A total of six Pixian broad-bean paste samples were obtained from Chengdu, including sample 1 (Sichuan Province Dandan Condiment Co. Ltd., Chengdu, China), sample 2 (Sichuan Pixiandouban Co. Ltd., Chengdu, China), sample 3 (Sichuan Gaofuji Food Co. Ltd., Chengdu, China), sample 4 (Chuanjiao Branch Co. of Sichuan Youlian Seasoning and Foods Co. Ltd., Chengdu, China), sample 5 (Sichuan Chuanpidouban Co. Ltd., Chengdu, China) and sample 6 (Chengdu Wangfeng Food Co. Ltd., Chengdu, China). Fermentation period of all Pixian broad-bean paste samples was near one year. The samples were ground and stored at 4 °C until analysis.

All chemicals used in this study were of the analytical and chromatographic grade. Standard organic acids (oxalic acid, tartaric acid, citric acid, lactic acid) were purchased from Kelong chemical plant (Chengdu, China). Other standards such as succinic acid, ketoglutaric acid, fumaric acid and adipic acid were purchased from Sigma-Aldrich (St. Louis, MO, USA), while malic acid was purchased from Aladdin Bio-Chem Technology Co. Ltd. (Shanghai, China). All amino acid standards were obtained from Seebio Biotechnology Co. Ltd. (Shanghai, China). For amino acid analysis in the automatic amino acid analyzer, ion exchange column was pre-standardized using all amino acid standards.

### 3.2. Preparation of Standard Solution

Solutions of the individual organic acid standard and mix standard solution were prepared separately. Referring to some fermented bean paste articles, organic acids including nine were detected. Standard solution of organic acids (oxalic acid, succinic acid, fumaric acid, lactic acid and ketoglutaric acid) was prepared at 0.5 g/L, standard solution of organic acids (tartaric acid, malic acid) was prepared at 1 g/L, and standard solution of organic acids (adipic acid, citric acid) was prepared at 2 g/L concentration in 10 mL distilled water. The standard solution of amino acid was prepared according to GB/T 30987-2014 [34]. All standard solutions were stored at 4 °C until the further use.

### 3.3. Measurement of Non-Volatile Organic Acids

To measure the contents of non-volatile organic acidsin six Pixian broad-bean paste samples, a previous method of Shruti Shukla [14] was adopted. Briefly, 2.5 g of each sample was vortex stirred with 20 mL of ethanol (72%, *v*/*v*) for 1 min (MVS-1 spiral mixer, Beijing, China) and supersonic extracted for 30 min at 37 °C (KH3200E ultrasonic cleaners, Kunshan, China), and then centrifuged at 3500 rpm/min for 10 min at 4 °C (TDZ5-WS centrifuge, Shanghai, China). The residue was again extracted twice with 15 mL and 10 mL respectively of 72% ethanol and centrifuged. Each supernatant was collected in a 50 mL volumetric flask, anddiluted with 72% ethanol to volume. Extracted components were purified by chromclean™ SAX (SWELL, Chengdu, China) 500 mg/6 mL (reactivated with 3 mL of methanol and 3 mL of distilled water). Take 5 mL of the collection solution in the SAX column until it slowly shed, and discard the effluent. The column was then rinsed with 5 mL of distilled water and eluted with 5 mL of hydrochloric acid solution (20%, *v*/*v*), controlling the flow rate at 1 mL/min to 2 mL/min. The eluate was passed through a 0.45 μm filter and placed in a vial.

### 3.4. Measurement of Amino Acids

Measurement of amino acids contents in six Pixian broad-bean paste samples was extracted with distilled water. 2.5 g of each sample was supersonic extraction with 100 mL distilled water for 30 min at 35 °C (KH3200E ultrasonic cleaners, Kunshan, China), and then centrifuged at 10,000 rpm/min for 15 min at 4 °C (TDZ5-WS centrifuge, Shanghai, China). The supernatant was passed through a 0.45 μm filter and placed in a vial [34].

### 3.5. Chromatographic Conditions

#### 3.5.1. HPLC Equipment and Conditions

Qualitative and quantitative analysis of non-volatile organic acidsin Pixian broad-bean paste was carried out through HPLC (Waters, Suzhou, China), using Waters HPLC separation module (e-2695) coupled with Photodiode Array Detector (2998) and an Aminex HPX-87H Ion Exclusion column (300 mm × 7.8 mm, 3 μm). The 0.005 mol/L sulfuric acid solution was used as mobile phase with a flow rate of 0.5 mL/min, and the column temperature was 60 °C. The injection volume was 20 μL, and wavelength was 210 nm. The concentration of non-volatile organic acidsin Pixian broad-bean paste samples was determined by the peak area of standard samples.

#### 3.5.2. Amino Acid Automatic Analyzer Equipment and Conditions

Qualitative and quantitative analysis of amino acids in Pixian broad-bean paste was carried out through the amino acid automatic analyzer, a previous method of GB/T 30987-2014 was adopted, using an amino acid automatic analyzer (L-8900, Hitachi, Japan) and an ion exchange column (PF 4.6 mm × 60 mm). The mobile phase was with a flow rate of 0.35 mL/min, and the column temperature was 135 °C. The reaction liquid flow rate was 0.3 mL/min, wavelength decreased to 440 nm from 570 nm, and the injection volume was 20 μL. The concentration of amino acids in Pixian broad-bean paste samples was determined by the peak area of standard samples.

### 3.6. Sensory Evaluation

Quantitative descriptive sensory analysis was applied for evaluation of the Pixian broad-bean paste samples, using a ten-point interval scale (0 = none, 9 = extremely strong). The sensory evaluated panel was made of 15 females and 5 males, 20–30 years old, trained according to GB/T 16291.1-2012 [35]. Sensory sessions took place in a sensory laboratory, which complied with international standards for test room [36]. From the discussion and pre-experiment with the panel, the reference materials of taste and mouth feel were as follows: sour (0.03% citric acid), sweet (1% glucose), bitter (0.9% quinine sulphate), salty (1% salt), savory (0.2% sodium glutamate). Sensory evaluation was performed in coded, tasting cup containing 20 mL water with 1 gram samples. Samples were presented in a random order [36]. 

### 3.7. Statistical Analysis

Samples were tested in triplicate for each Pixian broad-bean paste and results were expressed as the mean value ± standard deviation (S.D.). The triplicate samples were statistically analyzed using SPSS 17.0 for Windows (SPSS Statistics, Chicago, IL, USA) via analysis of variance (ANOVA) and Student-Newman-Keuls tests (S-N-K). The differences were recognized as significant at *p* < 0.05. PLSR analysis was used to explore the relationship between Pixian broad-bean paste samples, sensory data and taste-active compounds of 6 samples through UNSCRAMBLER 9.7 (CAMO ASA, Oslo, Norway). 

## 4. Conclusions

In this study, non-volatile organic acids, and amino acids in Pixian broad-bean paste were qualitatively and quantitatively analyzed by HPLC and amino acid automatic analyzer, respectively. It was found that the fermentation process produced various organic acids and amino acids, and types and contents may vary depending on the sample origin. There were eight organic acids and eighteen amino acids found in six samples in this investigation, including fumaric acid which was first detected in fermented bean pasts. For instance, the flavor compounds of citric acid, glutamic acid, methionine and proline might be the most important flavor compounds for evaluating the quality and further assessment of Pixian broad-bean paste.

## Figures and Tables

**Table 1:** Retention times and regression equation of organic acids separated by HPLC analysis.

Organic Acids	Retention Time (min)	Regression Equation	Coefficient of Determination (R^2^)
Oxalic acid	7.7	Y = 578960x − 310149	0.9988
Ketoglutaric acid	8.2	Y = 424580x − 67687	0.9998
Citric acid	9.3	Y = 258516x − 1317.7	0.9999
Tartaric acid	10.0	Y = 185717x − 3217.2	0.9998
Malic acid	11.1	Y = 81471x − 4245.2	1.0000
Succinic acid	13.2	Y = 34040x − 3970.7	0.9999
Lactic acid	14.9	Y = 90125x − 79730	0.9980
Fumaric acid	15.7	Y = 6187179.8x + 55173.5	0.9949

**Table 2:** Analysis of organic acid contents of Pixian broad-bean paste samples (*n* = 3).

Organic Acids (mg/g)	Sample 1	Sample 2	Sample 3	Sample 4	Sample 5	Sample 6
Oxalic acid	1.7374 ± 0.023 ^b^	2.2211 ± 0.180 ^a,b^	2.0544 ± 0.220 ^a,b^	1.8466 ± 0.235 ^a,b^	2.0358 ± 0.221 ^a,b^	2.4724 ± 0.427 ^a^
Ketoglutaric acid	ND ^a^	ND ^a^	ND ^a^	ND ^a^	ND ^a^	0.3339 ± 0.135 ^b^
Citric acid	4.8283 ± 0.922 ^a,b^	6.3 ± 0.170 ^a^	5.1342 ± 0.154 ^a,b^	5.2523 ± 0.360 ^a,b^	4.1978 ± 0.394 ^b^	4.3645 ± 0.375 ^b^
Tartaric acid	0.2551 ± 0.001 ^a^	0.3896 ± 0.084 ^a^	0.261 ± 0.164 ^a^	0.1893 ± 0.110 ^a^	0.1957 ± 0.166 ^a^	1.3544 ± 1.354 ^b^
Malic acid	3.6491 ± 0.190 ^b^	3.0998 ± 0.362 ^a,b^	2.7515 ± 0.462 ^ab^	2.162 ± 0.500 ^a^	2.3646 ± 0.883 ^a,b^	2.797 ± 0.330 ^a,b^
Succinic acid	0.4142 ± 0.385 ^a^	0.2499 ± 0.144 ^a^	0.5556 ± 0.226 ^a^	0.1031 ± 0.098 ^a^	0.0976 ± 0.710 ^a^	0.9397 ± 0.066 ^b^
Lactic acid	0.4107 ± 0.114 ^b^	0.0925 ± 0.075 ^a^	0.1078 ± 0.090 ^a^	0.1065 ± 0.095 ^a^	0.3859 ± 0.125 ^b^	0.1026 ± 0.095 ^a^
Fumaric acid	0.0052 ± 0.002 ^d^	0.0022 ± 0.002 ^c^	0.0038 ± 0.001 ^d^	0.0005 ± 0.0004 ^a^	1.974 × 10^−5^ ± 10 × 10^−5 a,b^	0.0008 ± 0.0006 ^a^
Adipic acid	ND ^a^	ND ^a^	ND ^a^	ND ^a^	ND ^a^	ND ^a^
Total	11.3000 ± 1.432 ^b^	12.3550 ± 0.906 ^a^	10.8680 ± 1.330 ^b^	9.2781 ± 1.516 ^c^	9.6597 ± 1.287 ^b,c^	12.3643 ± 0.934 ^a^

ND: not detected values with different superscript roman letters (a–d) in the same row are significantly different (*p* < 0.05).

**Table 3:** The group of amino acids of different taste.

	Taste	Amino Acid
1	Sweet, Savory	Threonine (Thr), Serine (Ser), Glycine (Gly), Alanine (Ala), Proline (Pro)
2	Savory, Sour	Glutamine(Gln), Glutamic acid (Glu), Aspartic acid (Asp), Asparagine (Asn)
3	Bitter, Sweet	Lysine (Lys), Histidine (His), Arginine (Arg)
4	Bitter	Methionine (Met), Isoleucine (Ile), Leucine (Leu), Valine (Val), Tyrosine (Tyr), Phenylalanine (Phe)
5	Salty	Cysteine (Cys)

**Table 4:** Analysis of amino acid contents of Pixian broad-bean paste samples (*n* = 3).

Taste	Amino Acid (mg/g)	Sample 1	Sample 2	Sample 3	Sample 4	Sample 5	Sample 6
Sweet Savory	Threonine (Thr)	0.675 ± 0.009 ^d^	0.607 ± 0.010 ^e^	0.877 ± 0.009 ^b^	0.678 ± 0.007 ^d^	0.768 ± 0.009 ^c^	1.473 ± 0.006 ^a^
	Serine (Ser)	0.826 ± 0.016 ^b^	0.680 ± 0.013 ^b^	1.097 ± 0.577 ^b^	0.715 ± 0.041 ^b^	0.934 ± 0.011 ^b^	1.940 ± 0.018 ^a^
	Glycine (Gly)	0.434 ± 0.007 ^d^	0.368 ± 0.007 ^e^	0.597 ± 0.004 ^b^	0.423 ± 0.018 ^d^	0.455 ± 0.005 ^c^	1.048 ± 0.010 ^a^
	Alanine (Ala)	0.959 ± 0.018 ^d^	0.936 ± 0.017 ^d^	1.336 ± 0.005 ^b^	1.103 ± 0.008 ^c^	1.089 ± 0.004 ^c^	2.578 ± 0.034 ^a^
	Proline (Pro)	2.170 ± 0.059 ^b^	1.311 ± 0.021 ^d^	1.597 ± 0.048 ^c^	1.098 ± 0.019 ^e^	1.644 ± 0.019 ^c^	2.820 ± 0.040 ^a^
	Total	5.063 ± 0.010 ^a^	3.902 ± 0.068 ^e^	5.505 ± 0.011 ^a^	4.018 ± 0.057 ^d^	4.891 ± 0.0.37 ^c^	9.858 ± 0.030 ^b^
Savory Sour	Glutamine (Gln)	ND ^a^	ND ^a^	ND ^a^	ND ^a^	ND ^a^	ND ^a^
	Glutamic acid (Glu)	2.696 ± 0.020 ^c^	1.794 ± 0.034 ^e^	2.951 ± 0.001 ^b^	1.832 ± 0.002 ^e^	2.590 ± 0.030 ^d^	4.447 ± 0.047 ^a^
	Aspartic acid (Asp)	1.722 ± 0.015 ^f^	1.799 ± 0.031 ^e^	2.760 ± 0.002 ^b^	2.337 ± 0.023 ^c^	2.171 ± 0.025 ^d^	4.980 ± 0.063 ^a^
	Asparagine (Asn)	2.042 ± 0.045 ^a^	0.407 ± 0.007 ^e^	0.723 ± 0.011 ^d^	0.771 ± 0.010 ^c^	1.483 ± 0.014 ^b^	0.775 ± 0.005 ^c^
	Total	6.460 ± 0.080 ^b^	4.000 ± 0.072 ^c^	6.434 ± 0.014 ^a^	4.940 ± 0.014 ^ab^	6.244 ± 0.068 ^b^	10.201 ± 0.106 ^d^
Bitter Sweet	Lysine (Lys)	1.014 ± 0.004 ^d^	0.832 ± 0.011 ^f^	1.356 ± 0.003 ^b^	0.882 ± 0.003 ^e^	1.081 ± 0.016 ^c^	2.286 ± 0.035 ^a^
	Histidine (His)	0.283 ± 0.004 ^c^	0.247 ± 0.006 ^d^	0.317 ± 0.005 ^b^	0.225 ± 0.004 ^e^	0.228 ± 0.001 ^e^	0.486 ± 0.001 ^a^
	Arginine (Arg)	1.213 ± 0.001 ^d^	1.035 ± 0.017 ^e^	1.480 ± 0.023 ^b^	0.850 ± 0.015 ^f^	1.350 ± 0.002 ^c^	2.705 ± 0.067 ^a^
	Total	2.510 ± 0.009 ^a^	2.115 ± 0.035 ^d^	3.153 ± 0.025 ^b^	1.957 ± 0.023 ^d^	2.659 ± 0.014 ^a^	5.478 ± 0.046 ^c^
Bitter	Methionine (Met)	0.116 ± 0.002 ^b^	0.066 ± 0.001 ^e^	0.083 ± 0.001 ^d^	0.003 ± 0 ^f^	0.108 ± 0 ^c^	0.183 ± 0.002 ^a^
	Isoleucine (Ile)	0.781 ± 0.007 ^d^	0.718 ± 0.01 ^2f^	1.094 ± 0.006 ^b^	0.735 ± 0.004 ^e^	0.814 ± 0.004 ^c^	2.143 ± 0.008 ^a^
	Leucine (Leu)	1.194 ± 0.014 ^cd^	1.022 ± 0.017 ^e^	1.698 ± 0.005 ^b^	1.168 ± 0.017 ^d^	1.227 ± 0.015 ^c^	3.266 ± 0.039 ^a^
	Tyrosine (Tyr)	0.638 ± 0.009 ^c^	0.550 ± 0.010 ^d^	0.813 ± 0.001 ^b^	0.562 ± 0.023 ^e^	0.617 ± 0.007 ^d^	1.359 ± 0.007 ^a^
	Phenylalanine (Phe)	0.782 ± 0.020 ^c^	0.673 ± 0.013 ^c^	1.064 ± 0.003 ^b^	0.738 ± 0.023 ^c^	0.782 ± 0.085 ^c^	1.859 ± 0.114 ^a^
	Valine (Val)	0.884 ± 0.045 ^d^	0.858 ± 0.013 ^d^	1.222 ± 0.049 ^b^	0.858 ± 0.018 ^d^	0.991 ± 0.003 ^c^	2.326 ± 0.051 ^a^
	Total	4.394 ± 0.105 ^d^	3.887 ± 0.069 ^b^	5.975 ± 0.045 ^a^	4.065 ± 0.086 ^b^	4.540 ± 0.023 ^a^	11.137 ± 0.124 ^b,c^
Salty	Cysteine (Cys)	ND ^a^	ND ^a^	ND ^a^	ND ^a^	ND ^a^	ND ^a^
	Total	ND ^a^	ND ^a^	ND ^a^	ND ^a^	ND ^a^	ND ^a^

ND: not detected values with different superscript roman letters (a–f) in the same row are significantly different (*p* < 0.05).
